# Evaluation of a fully automated “sample-to-result” PCR-based diagnostic test for detecting A/C/G Streptococci from throat swab specimens

**DOI:** 10.1128/spectrum.02099-25

**Published:** 2025-12-31

**Authors:** Sonja Suutari-Kontio, Vesa Mäki-Koivisto, Ilkka S. Junttila, Laura E. Savolainen

**Affiliations:** 1 NordLab Joint County Authority for Wellbeing Services, Oulu, Finland; 2 Research Unit of Biomedicine, University of Oulu 6370, Oulu, Finland; 3 Faculty of Medicine and Health Technology, Tampere University 101287, Tampere, Finland; 4 Fimlab Laboratories 277009 , Tampere, Finland; Ann & Robert H. Lurie Children's Hospital of Chicago, Chicago, Illinois, USA

**Keywords:** *S. pyogenes*, *S. dysgalactiae*, throat, PCR

## Abstract

Group A Streptococcus (GAS) and group C/G Streptococcus (GCS/GGS) species cause bacterial pharyngitis in both adults and children. Occasionally, GAS can cause invasive and severe infections like septicemia, streptococcal toxic shock-like syndrome, and necrotizing fasciitis. Throat culture has remained the gold standard method for diagnosing GAS or GCS/GGS pharyngitis. Methodologically, bacterial cultivation is laborious and time-consuming. For this, nucleic acid-based tests have been developed since they are rapid and have high sensitivity and specificity compared to culture methods. However, only a few molecular methods for the detection of GAS and especially for GCS/GGS in clinical throat swab specimens have been described. This study compares the clinical and analytical performance of a fully automated “sample-to-result” PCR with the gold standard reference method, culture. According to our results, the sensitivity of pooled PCR for GAS, GCS, and GGS was 94.1%, and the specificity was 94.2%. Pooled positive predictive value was 80.0%, and the negative predictive value was 98.5%. These results highlight that PCR is a reliable method, especially for ruling out negative cases due to its high negative predictive value. However, its positive predictive value is lower, meaning there is a higher chance of false positives. A PCR-based method for the diagnosis of pharyngitis would speed up turnaround time (TAT) and automate laboratory processes, especially in locations far away from a central laboratory. Faster TAT with a sensitive and specific method could reduce unnecessary antibiotic use, since currently, clinicians often prescribe antibiotics empirically before final test results.

**IMPORTANCE:**

Pharyngitis is commonly caused by viruses or by Streptococci, the latter of which can be treated with antibiotics. Rapid differential diagnostics help to reduce unnecessary antibiotic use, as clinicians often initiate empirical treatment before culture results are available. In addition, a shorter turnaround time for laboratory diagnosis may prevent transmission or development of the more severe disease. Diagnosis of streptococcal pharyngitis is commonly done by culture, which requires at least 24 h incubation. The results of our study showed that the fully automated PCR system is a useful and reliable tool for speeding up the diagnosis and would be helpful, especially in locations far away from central laboratories.

## INTRODUCTION

*Streptococcus pyogenes* is a gram-positive, spherical (coccus) bacterium, typically arranged in chains. It is a member of the Lancefield group A Streptococcus (GAS), which completely lyses red blood cells when cultured on blood agar. GAS is an obligate human pathogen that naturally infects animals. In humans, it resides on the skin and mucosal surfaces, particularly the throat, and mainly causes benign, noninvasive infections like pharyngitis and impetigo. In rare cases, GAS can cause invasive and severe infections like septicemia, streptococcal toxic shock-like syndrome, and necrotizing fasciitis. Transmission is most often direct person-to-person spread through respiratory droplets (1, 2). Approximately 14% of adult pharyngitis and 25-37% pediatric pharyngitis is caused by GAS (3). *Streptococcus dysgalactiae* subsp*. dysgalactiae* and *S. dysgalactiae* subsp*. equisimilis* (GCS and GGS, respectively) were initially considered as animal pathogens, but nowadays it is known that they cause similar infections as GAS and share the same virulence factors, including M protein, streptolysin O, streptolysin S, and streptokinase (4, 5).

Throat culture is the gold standard for diagnosing GAS or GCS/GGS pharyngitis (6). There are also rapid antigen detection tests (RADTs) available, but they detect only *S. pyogenes*. RADT usually has high specificity (>95%) but reduced sensitivity (70%–90%) compared to the reference method (7). In Finland, clinical guidelines (Sore throat, Finnish Current Care Guidelines, 2020) recommend using RADT primarily, and throat cultures should be taken only if symptoms continue. However, during the epidemic, throat culture is necessary for the antibiotic resistance testing and tracing of asymptomatic carriers (8). This reference method requires throat sample culturing on a blood sheep agar and incubation at 24–48 h in a CO_2_ atmosphere at +37°C (3, 6). The fastest turnaround time for the reference method is 24 h, but especially in Northern Finland, where geographical distances are long, TAT can rise to several days. This practically means that a patient may already have recovered by the time the culture results arrive. Although streptococcal pharyngitis may often be self-limiting, treatment with appropriate antibiotics is known to reduce symptom severity and duration, decrease transmission of the organism, and reduce the risk of post-infections (4). For this, nucleic acid-based tests have been developed since they are rapid and have high sensitivity and specificity (>90% and >95%) compared to culture methods (9, 10, 11, 12, 13)

Both commercial and laboratory-developed molecular methods are available for detecting GAS from throat swab specimens, but molecular methods, especially PCR methods detecting all three major pharyngeal pathogens, GAS, GCS, and GGS, are not widely used and have been relatively scarcely studied (9, 10, 14–17). The aim of this study was to investigate whether a PCR-based device could replace throat culture in the diagnosis of pharyngitis and to evaluate its clinical and analytical performance compared to the reference method, plate culture, which is considered the gold standard. To our knowledge, there are no other studies comparing throat culture with the PCR method capable of detecting all three major pharyngeal pathogens from a throat sample.

## MATERIALS AND METHODS

### Study design

Totally, 346 throat swab specimens were collected in eSwab (Copan, Murrieta, CA, USA) during 2023–2024 from adult patients with sore throat, visiting primary health care units in Wellbeing Services Counties of North Ostrobothnia and Kainuu, Finland. After collection, the samples were sent to the microbiology laboratory in Oulu. Depending on where the sample was geographically collected, the estimated transfer time to Oulu varied from a few hours to 2 days. Upon arrival at the laboratory, plate culture was done, and the sample tube was frozen at −20°C until the PCR run was performed.

### Sample anonymization

All enrolled samples were anonymized by assigning a unique study number without any patient identifier (e.g., name, birth date, medical record number, etc.).

### Culture

Samples were cultured on blood agar plates containing Mueller Hinton agar (BD Difco, Franklin Lakes, NJ, USA), tryptone soya agar (Oxoid, Thermo Fisher Scientific Inc., Waltham, MA, USA), 5% sheep blood (Bio Karjalohja Oy, Karjalohja, Finland), colistin and oxalic acid (Oxoid Streptococcus Supplement, Thermo Fisher Scientific Inc., Waltham, MA, USA). The plates were incubated for 48 h in a CO_2_ atmosphere at +37°C, and the results were interpreted by trained laboratory personnel. β-Hemolytic colonies were identified by Vitek MS Maldi-Tof mass spectrometry (bioMérieux, Marcy-l'Étoile, France).

### NeuMoDx Strep A/C/G Vantage Assay

The samples were thawed to room temperature and, after vortexing, analyzed on the NeuMoDx 288 molecular system (Strep A/C/G Vantage Assay, NeuMoDx Molecular, Inc., Ann Arbor, MI, USA; Qiagen, Hilden, Germany) (in this report, ACG-PCR) according to the manufacturer’s instructions. The NeuMoDx Strep A/C/G Vantage Assay is a PCR-based method providing rapid *in vitro* qualitative results for the detection and differentiation of *S. pyogenes* and *S. dysgalactiae* (pyogenic groups C and G β-hemolytic Streptococcus, including subsp. *dysgalactiae* group C, and *Streptococcus dysgalactiae* subsp. *equisimilis* groups C and G) in throat swab specimens obtained from symptomatic patients. The results were interpreted as positive or negative by NeumoDx according to the manufacturer’s limit of detection (LOD): GAS, 50 cfu/mL; GCS, 2,500 cfu/mL; and GGS, 10,000 cfu/mL.

### Quality control

One external negative control (eSwab containing only liquid Amies broth) and one internal positive control were processed on the NeuMoDx system each day of testing during the study. Internal positive control was laboratory developed, including 10 µL of *S. pyogenes* (ATCC 19615), *S. dysgalactiae* subsp*. dysgalactiae* (T-57111), and *S. canis* (T-59013) in one eSwab. The included Sample Process Control (SPC1) needed to be “pass” for including sample data from a given day in the performance analysis.

### Discordant results

ACG-PCR test results that differed from the CDC gold standard throat culture method were regarded as discordant. For performance analysis, discrepancies were handled as follows: if the ACG-PCR test result was negative and the CDC reference method was positive, a false-negative result was assumed and included in the performance analysis. If the ACG-PCR test result was positive and the CDC reference method was negative, a false-positive result was assumed and included in the performance analysis. All discordant results were confirmed with the isothermal nucleic acid amplification method (Solana Strep Complete Assay, QuidelOrtho Corporation, San Diego, CA, USA) (in this report, ACG-NAAT).

### Analytical sensitivity

A total of 10 spiked control samples were used to evaluate the analytical sensitivity of the ACG-PCR test. GAS (*Streptococcus pyogenes*, ATCC 19615), GCS (*Streptococcus dysgalactiae* subsp. *dysgalactiae*, T-57111), and GGS (*Streptococcus canis*, T-59013) McFarland suspensions from 1 to 10^4^ cfu/mL were performed in an eSwab tube, and mixed concentrations for all combinations were tested. A serial dilution method was used, and the initial bacterial loads in an eSwab tube were 8.2 × 10^6^ cfu/mL for GAS, 6.8 × 10^6^ cfu/mL for GCS, and 1.7 × 10^6^ cfu/mL for GGS.

### Invalid results

All invalid results were indeterminate results, which usually indicates an instrument error. In that case, the test was repeated from the same sample aliquot automatically.

### Data analysis

Sensitivity, specificity, positive predictive value, negative predictive value, and 95% confidence intervals were calculated using MedCalc Software (MedCalc Software Ltd., Diagnostic test evaluation calculator. Confidence intervals are “exact” Clopper-Pearson confidence intervals. Available at https://www.medcalc.org/calc/diagnostic_test.php, version 23.2.1; accessed 22 May 2025). Microsoft Excel (Microsoft, Redmond, WA, USA) was used to create tables and figures.

## RESULTS

### Clinical performance characteristics of ACG-PCR

Twenty samples were culture positive for GAS out of 346 samples (5.8%), and 31/346 were ACG-PCR positive (9.0%). Results of the ACG-PCR test provided a sensitivity of 100%, accuracy of 96.3%, and specificity of 96.0% when compared to the reference method. The positive predictive value was 64.5%, and the negative predictive value was 100% (Table 1).

**TABLE 1:** Clinical performance of ACG-PCR for the detection of GAS and GCS/GGS compared to reference method^*a*^

	Sensitivity (*n*) (95% CI)	Specificity (*n*) (95% CI)	PPV (95% CI)	NPV (95% CI)	Accuracy (95% CI)
GAS	100 (20/20) (83.2–100)	96.0 (262/273) (92.9–98.0)	64.5 (50.5–76.4)	100 (98.6–100)	96.3 (93.4–98.1)
GCS/GGS	91.7 (44/48) (80.0–97.7)	98.1 (262/267) (95.7–99.4)	89.8 (78.6–95.5)	98.5 (96.2–99.4)	97.1 (94.7–98.7)
Pooled, GAS, and GCS/GGS	94.1 (64/68) (85.6–98.4)	94.2 (262/278) (90.8–96.7)	80.0 (71.2–86.6)	98.5 (96.2–99.4)	94.2 (91.2–96.4)

^*a*^ Sensitivity, specificity, positive predictive value (PPV), negative predictive value (NPV), accuracy, and 95% confidence intervals are presented as percentages.

Forty-four samples were culture positive for GCS/GGS out of 346 samples (12.7%), and 49/346 were ACG-PCR positive (14.2%). Results of the ACG-PCR test provided a sensitivity of 91.7%, accuracy of 97.1%, and specificity of 98.1% for the GCS/GGS target, when compared to reference method. The positive predictive value was 89.8%, and the negative predictive value 98.5% (Table 1).

Pooled sensitivity for GAS and GCS/GGS was 94.1%, with an accuracy of 94.2%, and a specificity of 94.2%. The positive predictive value was 80.0%, while the negative predictive value was 98.5% (Table 1).

### Comparison of the cycle threshold values for true positives and false positives

**Fig 1:**
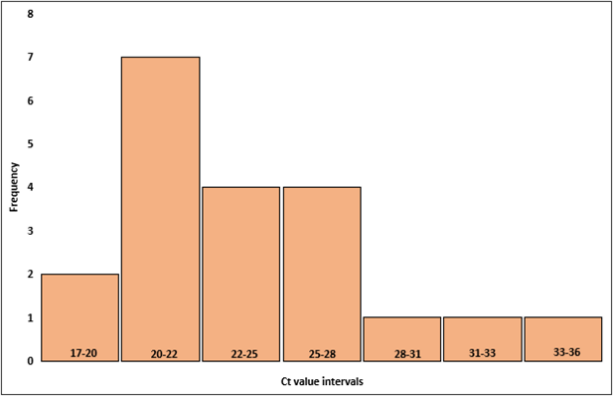
Distribution of Ct values in positive GAS samples by both ACG-PCR and culture (true positives). The histogram shows the frequency distribution of Ct values, and the bars represent the number of samples at each Ct value interval.

**Fig 2:**
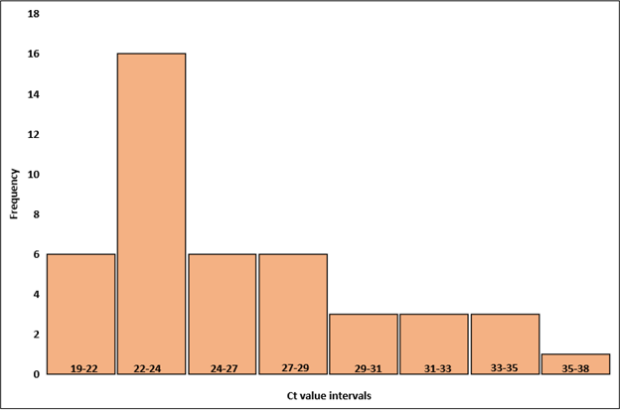
Distribution of Ct values in positive GCS/GGS samples by both ACG-PCR and culture (true positives). The histogram shows the frequency distribution of Ct values, and the bars represent the number of samples at each Ct value interval.

Sixty-four samples tested positive by both methods and were classified as true positives. Of these, 44 were GCS/GGS positive and 20 were GAS positive. Among these, the pooled mean of ACG-PCR cycle threshold (Ct) value was 25.4, with a pooled median of 24.4. The Ct values ranged from 17.2 to 38.1. For true GAS positives, mean Ct value was 24.6, and the median was 23.8. The Ct values ranged from 17.2 to 36.6 (Fig. 1). For true GCS/GGS, mean Ct value was 25.8, and the median was 24.4. The Ct values ranged from 19.5 to 38.1 (Fig. 2). One sample tested positive for GAS by culture, while PCR detected both GAS and GCS/GGS, with Ct values of 25.8 and 33.0, respectively.

**Fig 3:**
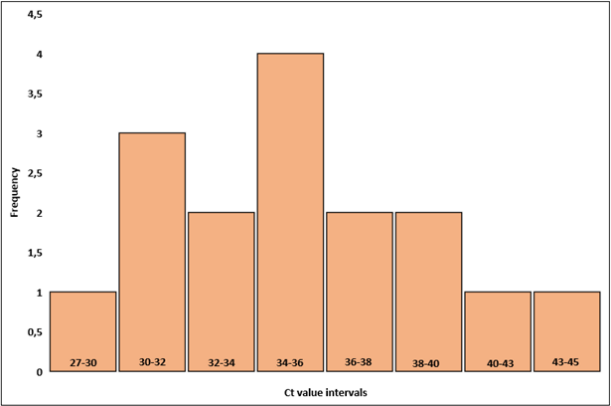
Distribution of Ct values in false-positive samples. The histogram shows the frequency distribution of Ct values from throat swab samples that tested positive for pharyngeal pathogens by PCR but negative in culture. The bars represent the number of samples at each Ct value interval.

Additionally, 16 samples (11 GAS and 5 GCS/GGS) tested negative by culture but positive by ACG-PCR, classified as false positives. For ACG-PCR GAS positives, mean Ct value was 35.1, and the median was 35.2. The range was 27.5–45.5. For ACG-PCR GCS/GGS positives, mean Ct value was 36.8, and the median was 38.7. The range was 30.9–40.6. When looking at pooled results for false-positive samples, the mean Ct value was 35.6, with a median of 35.3. The range was 27.5–45.5 (Fig. 3).

### Discordant results

All 20 samples with discordant results were analyzed with ACG-NAAT (16 false positives and 4 false negatives). Results are shown in Table 2. Seven out of eleven ACG-PCR GAS-positive and culture-negative samples were also positive in ACG-NAAT, while 4/11 samples were negative. When GCS/GGS ACG-PCR positives were analyzed, only 1/5 was positive also in ACG-NAAT, and four samples gave negative results. There were a total of four discordant samples with positive in culture but negative in ACG-PCR; all of those were GCS/GGS strains. When analyzed with ACG-NAAT, 3/4 were GCS/GGS positive and 1/4 was negative.

**TABLE 2:** The results of culture, ACG-PCR with Ct values, and ACG-NAAT for samples with discordant results^*a*^

Sample no.	Culture result	ACG-PCR result	Ct value	ACG-NAAT result
7	NEG	GAS+	37.73	NEG
9	NEG	GCS/GGS+	30.88	GCS/GGS+
45	NEG	GAS+	30.60	GAS+
71	NEG	GCS/GGS+	40.62	NEG
74	NEG	GCS/GGS+	35.09	NEG
85	NEG	GCS/GGS+	38.70	NEG
93	NEG	GAS+	35.42	GAS+
102	G/C+	NEG	–	GCS/GGS+
113	G/C+	NEG	–	NEG
154	NEG	GAS+	41.05	NEG
156	NEG	GAS+	32.95	GAS+
204	NEG	GAS+	33.20	GAS+
208	NEG	GAS+	35.23	GAS+
211	G/C+	NEG	–	GCS/GGS+
225	NEG	GAS+	45.47	NEG
228	NEG	GCS/GGS+	38.83	NEG
242	G/C+	NEG	–	GCS/GGS+
244	NEG	GAS+	27.48	GAS+
312	NEG	GAS+	35.37	NEG
348	NEG	GAS+	31.61	GAS+

^*a*^ Cells with no detected amplification are indicated as “–” to reflect non-detection.

### Analytical sensitivity of ACG-PCR

To analyze the sensitivity of PCR test, we utilized spiked bacteria samples. GAS, GCS, and GGS bacterial strains were mixed at varying concentrations, as shown in Table 3. ACG-PCR was able to distinguish different strains at the highest concentration, 10^4^ cfu/mL. When 10^4^ cfu/mL and 1 cfu/mL dilutions of GAS, GCS, and GGS were mixed, only 10^4^ cfu/mL concentrations gave positive results in all the mixed cases.

**TABLE 3:** Ct values for GAS and GCS/GGS targets across two PCR runs by sample tube^*a*^

		Ct value			
Tube	Strain (cfu/mL)	GAS, run 1	GAS, run 2	GCS/GGS, run 1	GCS/GGS, run 2
1	A + C + G (10^4^)	28.90	28.05	33.72	33.31
2	A + C + G (10^4^)	34.32	34.50	31.28	34.56
3	A + C + G (10^2^)	–	–	–	38.47
4	A + C + G (10^2^)	40.00	39.18	42.22	38.66
5	A + C + G (1)	–	–	–	–
6	A + C + G (1)	–	–	–	–
7	A (10^4^) + G/C (1)	35.10	34.08	–	
8	A (10^4^) + G/C (1)	34.10	34.28	–	–
9	A (1) + G/C (10^4^)	–	–	36.25	36.09
10	A (1) + G/C (10^4^)	–	–	35.44	35.69

^*a*^ This table presents the individual Ct values obtained for both GAS and GCS/GGS targets in two separate PCR runs (run 1 and run 2) when strains were mixed in one sample tube at varying concentrations. Cells with no detected amplification are indicated as “–” to reflect non-detection.

## DISCUSSION

Throat culture remains the gold standard method for diagnosing streptococcal pharyngitis. However, bacterial culture is a time-consuming and laborious method. In Northern Finland, similar to other places where microbiology laboratories may be centralized, and sample transport distances are long, test results can take 2–3 days. When test results reach the clinician, the opportunity for timely and targeted antimicrobial treatment is often lost. By then, the patient is either on the mend or already receiving an empirically chosen broad-spectrum antibiotic. For this, there is a need for a faster and less labor-intensive laboratory method to replace throat culture.

The comparison between the PCR test and traditional culture testing shows that PCR demonstrates strong performance in detecting positive and negative cases. According to our studies, pooled sensitivity was 94.1%, and the specificity was 94.2%. According to the manufacturer (4), sensitivity for GAS is 100%, and the specificity is 100%. In our study, sensitivity for GAS was 100%, and the specificity was 96.0%. Sensitivity for GCS/GGS is 95.9%, and the specificity is 100%, according to the manufacturer. In our study, sensitivity for GCS/GGS was 91.7%, and the specificity was 98.1%. A study comparing POC-PCR, RADT, and culture for detecting GAS had sensitivities of 95.5%, 85.5% and 71.8% and specificities were 99.3%, 93.7% and 100%, respectively (18).

These results suggest that ACG-PCR is a reliable method, especially for ruling out negative cases due to its high negative predictive value. However, its positive predictive value is lower, meaning there is a higher chance of false positives compared to false negatives. Mean Ct value was 35.6 for false-positive cases compared to true-positive cases which had a mean Ct value of 25.4. When analyzing false-positive results, these Ct values indicate that the detected positive results by ACG-PCR generally have high Ct values, suggesting a low bacterial load in these samples. ACG-PCR may detect bacterial DNA at very low concentrations, which the traditional culture method fails to identify. In addition, the presence of bacterial DNA without active, culturable bacteria might be due to previous infections, antibiotic treatment effects, or non-viable bacteria. While ACG-PCR is highly sensitive, some of these cases could be false positives due to background contamination or assay limitations.

The high Ct values suggest that the bacterial load in these samples is very low, potentially below the detection limit of culture. These findings highlight the increased sensitivity of ACG-PCR but also raise questions about the clinical significance of low-bacterial load detections. The data set consisted of throat samples from adults, and it is known that approximately 10% of children carry bacteria such as *S. pyogenes* asymptomatically in their throat (7). Therefore, the necessity of a throat sample should be carefully considered for each patient, and the device’s cut-off value must be set low enough to avoid false-positive results. The threshold must be sufficiently low, based on these data, around 35, to avoid false-positive results. Otherwise, there is a risk of unnecessary antibiotic prescriptions, as high Ct values are unlikely to indicate symptomatic disease. Additional analysis, such as clinical correlation or repeat testing, may be needed to determine the relevance of these results. In clinical applications, this could mean that additional confirmatory testing may be required for positive cases. On the other hand, the Ct range for real positives was 17.2–38.1. It should be noted that in our material, only 16 samples were false positives; so based on these data, we cannot determine a specific Ct cut-off.

When analyzing false-negative samples, there are several possible explanations. Mutations in the PCR target region can yield a false-negative result. If the bacteria have mutations in the genetic region targeted by the PCR primers, the test may fail to detect them, even though they grow in culture. Also, low DNA concentration in the sample can cause a false-negative result because PCR requires a sufficient amount of bacterial DNA for detection. If sample processing or storage has degraded the DNA, PCR may yield a false negative, even if viable bacteria are present in culture. Additionally, PCR inhibitors in the sample, e.g., hemoglobin, mucus components, or sample collection reagents, can interfere with PCR amplification, leading to false-negative results. PCR detects DNA, but it could be speculated that if bacteria are in a capsule form or not actively replicating, their DNA might not be sufficiently available in the sample, even though they grow well in culture. Different tests may analyze different parts of the sample. If the sample is not fully homogeneous, viable bacteria might be present in the cultured portion but not in the part used for PCR testing.

Additionally, all false-negative results were observed for GCS/GGS strains, for which ACG-PCR has a higher LOD than for GAS. This difference in limits of detection also became evident when testing spiked control strains. According to the manufacturer, ACG-PCR’s LOD is 50 cfu/mL for GAS, 2,500 cfu/mL for GCS, and 10,000 cfu/mL for GGS, indicating a considerable difference.

Analytical sensitivity was evaluated with spiked samples. At the lowest concentration (1 cfu/mL), neither GAS nor GCS/GGS assays yielded detectable amplification. Concentration of 100 cfu/mL was near the limit of detection, and 10^4^ cfu/mL yielded positive results from all tested pathogens and combinations. For tubes 2 and 4, the difference in GCS/GGS Ct values was the greatest between run 1 and 2, most likely due to the small number of bacteria in the tube. In both cases, the bacterial load is close to the manufacturer’s stated LOD value (cfu/mL). These results are in line with the LODs provided by the manufacturer.

All samples that showed discrepancies between culture and PCR tests were reanalyzed using the ACG-NAAT system, which is based on isothermal nucleic acid amplification. This method operates differently from PCR but still detects specific genetic regions in *S. pyogenes* and *S. dysgalactiae* DNA. In multiple cases where ACG-PCR is positive, ACG-NAAT remains negative. ACG-NAAT appears to have lower sensitivity than ACG-PCR, possibly missing some true positives. In some cases, ACG-NAAT detects a positive result while ACG-PCR is negative. In our case, 4 out of 346 were false-negative results, and when analyzed with ACG-NAAT, 3 out of 4 were positives. This suggests that ACG-PCR’s false-negative results are likely due to a mutation in the genetic region recognized by its primers. ACG-NAAT likely targets different genetic regions in Streptococci compared to ACG-PCR, which would explain the differences in results.

A PCR-based method for throat diagnostics would speed up result TATs, especially in Northern Finland, where distances are long. With the ACG-PCR device, results can be obtained in under 2 h without sample preprocessing. Transitioning from throat culture to a PCR-based method, either partially or entirely, would free up personnel resources for other tasks.

Finally, this study also had some limitations; for example, we did not assess detailed TAT, since we used frozen samples. In addition, we did not define an exact Ct cut-off value for ACG-PCR due to the small sample size (16 false positives) and the lack of clinical patient data, such as exact symptoms and used antibiotics.

### Conclusion

PCR methods might be useful tools for the diagnosis of streptococcal pharyngitis. The ACG-PCR method investigated in this study showed reliable performance compared to the reference, culture method. Further studies, particularly in estimating an optimal cut-off, are warranted. Another important open question regarding bacterial PCR methods in throat specimens is the differentiation between carrier and infection. Practically, this underlines the need for careful clinical estimation in concert with appropriate laboratory confirmation. From the point of view of the clinical laboratory, the choice of method for routine clinical use needs to balance accuracy of the method, the TAT, laboratory workflow, and overall costs. Obtaining full automation with rapid results in diagnostics, with appropriate use of the test, brings a clear advantage as compared to the culture, including shorter TAT and reduced use of unnecessary antibiotics.
